# Native and Bioengineered Exosomes for Ischemic Stroke Therapy

**DOI:** 10.3389/fcell.2021.619565

**Published:** 2021-03-23

**Authors:** Haroon Khan, Jia-Ji Pan, Yongfang Li, Zhijun Zhang, Guo-Yuan Yang

**Affiliations:** ^1^Med-X Research Institute, School of Biomedical Engineering, Shanghai Jiao Tong University, Shanghai, China; ^2^Department of Neurology, Ruijin Hospital, School of Medicine, Shanghai Jiao Tong University, Shanghai, China

**Keywords:** brain ischemia, extracellular vesicles, stroke, exosomes, bioengineered exosomes

## Abstract

Exosomes are natural cells-derived vesicles, which are at the forefront toward clinical success for various diseases, including cerebral ischemia. Exosomes mediate cell-to-cell communication in different brain cells during both physiological and pathological conditions. Exosomes are an extensively studied type of extracellular vesicle, which are considered to be the best alternative for stem cell–based therapy. They can be secreted by various cell types and have unique biological properties. Even though native exosomes have potential for ischemic stroke therapy, some undesirable features prevent their success in clinical applications, including a short half-life, poor targeting property, low concentration at the target site, rapid clearance from the lesion region, and inefficient payload. In this review, we highlight exosome trafficking and cellular uptake and survey the latest discoveries in the context of exosome research as the best fit for brain targeting owing to its natural brain-homing abilities. Furthermore, we overview the methods by which researchers have bioengineered exosomes (BioEng-Exo) for stroke therapy. Finally, we summarize studies in which exosomes were bioengineered by a third party for stroke recovery. This review provides up-to-date knowledge about the versatile nature of exosomes with a special focus on BioEng-Exo for ischemic stroke. Standard exosome bioengineering techniques are mandatory for the future and will lead exosomes toward clinical success for stroke therapy.

## Introduction

Stroke is a leading cause of death and neurological disability, and it has no effective treatment up to now (Li et al., 2020a; McCann and Lawrence, 2020). Approximately 6.7 million people die due to stroke each year (Cunningham et al., 2020), and 87% of these deaths are due to ischemic stroke (Dabrowska et al., 2019). Pathophysiological responses after stroke are complex, and presently, no better choice is available for stroke treatment except tissue plasminogen activator (Otero-Ortega et al., 2019; Pan et al., 2020), the only useful drug within 4–6 h of clearly defined symptom onset (Malone et al., 2019; Wang J. et al., 2020). However, due to its narrow therapeutic window, less than 5% of patients benefit from it (Wang et al., 2018). Even though there is some progress in stroke treatment, current therapeutic concepts are limited, and development of novel therapy for stroke survivors is critical.

Since the discovery that mesenchymal stem cells (MSCs) could produce new neurons (Sanchez-Ramos et al., 2000), much improvement has been done in MSC research (Kruminis-Kaszkiel et al., 2020). MSCs are multipotent, self-renewing exogenous cell populations present in adults as well as developing individuals and can differentiate into neuron as well glial lineages (Bai et al., 2020). However, with the advancement of this field, scientists have found that MSCs can have unpredictable consequences for target as well non-target organs (Wei et al., 2019). MSC implantation in the brain is invasive and can cause damage to healthy tissue, and they have poor survival in hypoxic and inflammatory conditions (Kim H.Y. et al., 2020). MSCs are big (15–40 μm), which makes them easily get trapped in small-diameter vessels, causing vascular occlusion and a decrease in cerebral blood flow (Bang and Kim, 2019). Hence, MSC delivery to the ischemic region is difficult, and this limits their clinical use for the treatment of ischemic stroke. While considering the therapeutic abilities of stem cells, researchers are shifting their interest toward stem cell–derived products, one of which is the exosome.

Exosomes are cell membrane–derived vesicles secreted by almost all body cells (Wang S. et al., 2020). The secretion process of exosomes is related to the mechanism of waste disposal. Wolfers et al. (2001) found that exosomes were an important source of intercellular communication in cancer. However, now it is confirmed by numerous studies that exosomes mediate communication by delivering DNA, RNA, lipids, and proteins in between cells and tissues (van Niel et al., 2018; Lin et al., 2020), playing an important role in controlling the biological expression of the recipient cells and leading to the activation of their signaling pathways (Cheng et al., 2019).

Exosomes are the best choice because of their natural characteristics, such as negligible toxicity, circulation stability, production and storage advantages, ability to encapsulate endogenous bioactive molecules, strong protection for cargo, and excellent transport efficacy to distant body cells, specifically for brains cells as they can pass through the blood–brain barrier (BBB) easily (Rufino-Ramos et al., 2017; Bang and Kim, 2019). Stem cell–derived exosomes’ beneficial effects were studied in animal models of stroke, traumatic brain injury, Alzheimer’s disease, status epilepticus–induced brain injury, multiple sclerosis, and spinal cord injury (Kodali et al., 2020). Exosomes are the most promising therapeutic avenues capable of addressing the need for regenerative and therapeutic treatment deficiencies for stroke patients (Webb et al., 2018; Gharbi et al., 2020). In addition, synthetic therapeutic agents may suffer from immunogenicity and toxicity, but the origin of exosomes is biological, that is why they are less likely to induce adverse effects (de Abreu et al., 2020).

Exosomes’ ability to cross the BBB makes them a suitable candidate for brain diseases as well as increasing interest in utilization of exosomes as drug delivery systems to the brain (Wood et al., 2011). The BBB is composed of brain macrovascular endothelial cells (BMECs), pericytes, astrocytes, and tight junctions, which allow selective transport of some compounds while inhibiting entry of toxic substances in between the blood and brain (Ballabh et al., 2004). The exosome BBB crossing mechanism remains unclear; however, research examining exosome trafficking through the BBB points toward absorption by BMECs via endocytosis, where they fuse to BMEC endosomes and are released to the brain (Chen et al., 2016; Lu et al., 2020). Interaction between exosomes and BMECs *in vitro* shows that, in healthy and stroke-like conditions, exosomes retain their BBB crossing ability (Chen et al., 2016). Exosomes crossed the BBB and were taken up by endothelial cells in a 3-D BBB static model (Jakubec et al., 2020). This finding of exosome BBB crossing has been confirmed by a number of studies (Grapp et al., 2013; Long et al., 2017; Yuan et al., 2017; Qu et al., 2018) however, biodistribution studies of exosomes reveal that, after intravenous administration of native exosomes to the body, they were rapidly cleaned from the target site and accumulated in organs of the reticuloendothelial system, such as the lungs, liver, and spleen, and very few exosomes were found at the target site (Lai et al., 2014; Wiklander et al., 2015; Tian et al., 2018; Lu and Huang, 2020). Hence, there is need to engineer exosome targeting characteristics before its use as a therapeutic agent for stroke therapy.

Exosome research is still in its infancy, particularly in central nervous system (CNS) diseases; therefore, a better understanding of exosomes is of formidable importance to optimally utilize them for therapeutic purposes. Even though the potential therapeutic abilities of exosomes are considered to be well-established fact now, several challenges still need to be addressed before its successful clinical translation. These include the processes by which they are formed and their function, exosome targeting and trafficking, internalization, dose optimization, route of administration, and strategies for modification of exosomes to steer their delivery toward their target sites of action. Here, first we overview the exosome process of biogenesis, composition, and uptake by cells. Next, we survey some of the latest discoveries that add unexpected new twists to our understanding of exosome function and potential for stroke therapy. Furthermore, we highlight exosomes’ versatile nature as being the best fit for brain targeting and review the methodologies of exosome engineering for brain targeting. Finally, we focus on and summarize the studies in which exosomes were bioengineered by a third party for stroke therapy and discuss how this field could advance. In this review, we use the term “exosomes” for some studies on behalf of extracellular vesicles (EVs) (Chivet et al., 2014; Liu et al., 2019; Tieu et al., 2020).

## Overview of Exosome Biogenesis

**FIGURE 1:**
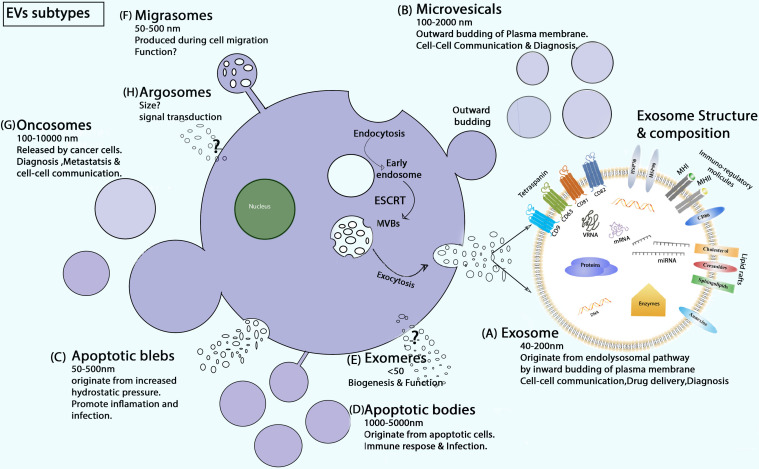
Schematic of EV biogenesis and subtypes. **(A)** Exosome secretion (ESCRT dependent) and composition. **(B)** MVs are formed during inflammatory and hypoxic conditions as buds off of the plasma membrane. **(C)** Apoptotic blebs are EVs that generate with increased cell contraction and hydrostatic pressure (Xie et al., 2020). **(D)** Apoptotic bodies are released only during programmed cell death (Jadli et al., 2020). **(E)** Exomeres are recently discovered EVs, and their biological function and biogenesis is yet unknown (Zhang et al., 2018; Xie et al., 2020). **(F)** Migrasomes are oval-shaped EVs produced during cell migration. **(G)** Oncosomes are membrane-derived large and small EVs released by cancer cells. They contain a unique signature of the tumor cells from which they are secreted (Chuo et al., 2018). **(H)** Argosomes are exosome-like vesicles, and their biogenesis starts from the basolateral membrane of Drosophila discoid cells and can take part directly in the transferring of molecules from producer to recipient cells (Borges et al., 2013).

Exosomes were first reported by Trams et al. (1981), Xin et al. (2014); Witwer and Théry (2019). They were discovered in *in vitro* cell culture of sheep red blood cell supernatants with structural size ranging from 40 to 200 nm (Yu et al., 2020). Exosomes are the most important subtype of EVs, whereas the literature also contains other EV subtypes, including microvesicles, apoptotic bodies, blebs, exomeres, migrasomes, oncosomes, and argosomes classified by size, biogenesis, origin, content, and function (Ma et al., 2015; Xie et al., 2020) (Figure 1). Exosomes differ from other types of EVs in the context of biogenesis, diameter, and contents.

Exosome biogenesis occurs from the endo-lysosomal pathway, in particular, when inward budding of the plasma membrane happens. Biogenesis starts with the development of early endosomes (EEs), then intraluminal vesicles (ILVs) in intracellular multivesicular bodies (MVBs), transport of MVBs to the plasma layer, and union of MVBs with the plasma membrane. The first step in the formation of EEs is budding from the plasma membrane and the fusion of primary endocytic vesicles. The *trans*-Golgi network and endoplasmic reticulum could promote EE formation and content. In the conversion process of EEs to MVBs, Rab5 and its effector VPS34/p150 act as important regulators (Huotari and Helenius, 2011). The most important protein playing a role in ILV formation is the endosomal-sorting complex essential for transport (ESCRT) protein family. This protein family includes four complexes, ESCRT-0, ESCRT-I, ESCRT-II, and ESCRT-III (Murphy et al., 2019; Kalluri and LeBleu, 2020). ILVs can also generate through the ESCRT-independent pathway. For the ESCRT-independent way of maturation, tetraspanin and CD63 are important factors. Neutral sphingomyelinase 2 enzyme is another important mediator of ESCRT-independent ILV maturation. This enzyme synthesizes the lipid ceramide that makes microdomains on the surface of MVBs, a process that is vital for ILV production (Murphy et al., 2019). Thus, MVB biogenesis can be ESCRT-dependent or -independent. In ESCRT-dependent biogenesis, MVBs take two paths, influenced by the protein ubiquitin checkpoint (Vietri et al., 2020). On the one hand, MVBs bind to lysosomes or autophagosomes in the cells and are degraded by the ubiquitinated cargo. On the other hand, MVBs are mediated by specific components of the actin, cortactin, microtubule skeleton, and Rab family (such as Rab27A and Rab27B), which are continuously transported and eventually stuck to the plasma membrane to secrete exosomes (Ostrowski et al., 2010; Villarroya-Beltri et al., 2016).

Through the biogenesis of exosomes, a series of molecular contents with cell biological activity selectively encapsulate into exosomes (Xu et al., 2018). The contents of exosomes can reflect the structure of donor cells (Valadi et al., 2007). Research indicates that exosomes are rich in many molecular substances, such as mRNA, miRNA, and other non-coding RNA (Vojtech et al., 2014; Xie et al., 2019); double-stranded DNA and mtDNA (Guescini et al., 2010; Sansone et al., 2017; Yang S. et al., 2017; Tsai et al., 2018); cholesterol, sphingolipids, and ceramides (Skotland et al., 2017); heat shock cognate proteins (HSP70, HSP90), adhesion molecules, and cell skeleton proteins (Muller, 2020); and receptors MHC-II, MHC-I, CD86 and tetraspanin proteins CD9, CD63, CD81, CD82, and enzymes (Thery et al., 2002; Pegtel and Gould, 2019). Once exosomes are grabbed by the recipient cell, they can release their exosomal cargo to the target cell performing different kinds of pathological and physiological functions.

## Mechanism of Exosome Uptake

Different uptake pathways involve exosome internalization by recipient cells. Exosome uptake routes are diverse and depend on both the donor and recipient cell type (Murphy et al., 2019). Researchers indicate that exosomes biologically resemble retroviruses as they share many properties. Both exosomes and retroviruses have comparable diameter of about 120 nm (Mathieu et al., 2019), are coated with a lipid membrane carrying genetic material originating from endosomal pathways (Margolis and Sadovsky, 2019), and trigger a specific reaction through their molecular contents in the recipient cells (Nolte-’t Hoen et al., 2016). The ability of exosomes to avoid a degradative pathway was revealed to resemble the route of human immunodeficiency virus (HIV) mechanism of cell uptake (Izquierdo-Useros et al., 2009). HIV causes infection by combining with the plasma membrane; likewise exosomes are taken up by recipient cells after combining with plasma membranes. However, the cells take up HIV virions through pathways, including multiple modes of macrophage proliferation, phagocytosis, and endocytosis (Melikyan, 2014). On the other hand, exosomes can also be taken up by different pathways, such as caveolin-mediated endocytosis, clathrin-mediated endocytosis, lipid raft-mediated endocytosis, macro-pinocytosis, and phagocytosis (Mulcahy et al., 2014; Murphy et al., 2019).

Even though exosomes have similarities to viruses in their uptake mechanism, there are some differences as well. In some cases, the uptake of exosomes is more complicated than viruses. For example, exosomes are abundant in macro-molecules rich in PS receptors, lectins, glycans, integrins, and other cell adhesion molecules. These allow exosomes to bind to and be taken up by nearby or faraway recipient cells via ligand-receptor interaction to release the substances they carry (van Dongen et al., 2016; Joshi et al., 2020). In summary, exosome uptake can be expressed in three ways: direct fusion with cell membranes, endocytosis, and ligand-receptor interaction. Other exosome surface proteins, such as CD9 and CD81, are included in cell–cell fusion, but whether they are included in the mediation of exosome–cell fusion lacks evidence, and further studies are needed to confirm.

## Insight of Native Exosomes for Stroke

Exosomes are nano-sized extracellular membrane vesicles that can work as a remedy for inflammation and improve functional and behavioral recovery in stroke models of rodents (Go et al., 2020).

### Stem Cell–Secreted Exosomes in Stroke Treatment

Among various stem cell types, MSCs are vastly studied cells (Kabat et al., 2020). MSCs were believed to treat various disease conditions by differentiating into healthy cells and regaining functionality, but later on, researchers found that this was because of the paracrine effect of MSCs on the surrounding host cells (Murphy et al., 2019; Hur et al., 2020). The paracrine signals of MSCs are due to exosomes (Maacha et al., 2020). Nowadays, it is proven by multiple research groups that exosomes contain various types of bioactive molecules possessing the properties and contents of their origin cell (Riazifar et al., 2019; Hur et al., 2020). Stem cell exosomes have been isolated, characterized, evaluated, and designed for enhancing beneficial effects in brain injury and neurodegenerative disease (Upadhya and Shetty, 2019). MSCs exosomes (MSCs-Exo) mediate secretion of cell waste to extracellular fluid and transmit it in between producer and target/recipient brain cells (Glebov et al., 2015; Zhang Z.G. et al., 2019). Under preclinical and clinical research, stem cell–derived exosome-based approaches have been verified as a promising regenerative medical treatment for ischemic stroke (Table 1).

**TABLE 1:** Native exosomes for ischemic stroke.

Exosomes used	Animal model	Target	Results	References
M2-Exo	Mouse	Stroke treatment	M2-Exo treatment improve neuronal survival, reduce infract volume, improve behavior deficit, Downregulated USP-14 Gene	Song et al., 2019
MSCs-Exo	Mouse model/*in vitro*	Hypoxic ischemia	Exosome treatment decreases neuron apoptosis and neuroinflammation while upregulating miR-21a-5p levels	Xin et al., 2020
MSCs-Exo	Rats	Stroke recovery	Promote neuroplasticity, functional recovery, inhibiting PTEN expression and upregulation of Exo-miR-17-92	Xin et al., 2017
hMSCs-Exo	Mouse Model of EAE	Treat MS	Reduce demyelination, decrease neuroinflammation, upregulated CD4 + CD25 + FOXP + Tregs	Riazifar et al., 2019
hMSCs-sEVs	Mouse	Ischemic stroke	Neuroprotective by depleting PMNs, e.g., monocytes, lymphocytes, and reverse post ischemic lymphopenia.	Wang C. et al., 2020
EPC-Exo	Mouse	Ischemic stroke	Decreases infract volume, NDS, apoptosis, Upregulate microvessel density miR-126, BDNF, p-TrkB/TrkB and p-Akt/Akt	Wang J. et al., 2020
MSCs-Exo	Monkey	Cortical injury	Motor function recovery and neurological dysfunction recovery by shifting inflammatory microglia toward anti-inflammatory.	Moore et al., 2019; Go et al., 2020
MSCs-Exo	Rats/*in vitro*	Brain Ischemia	Improved motor function, anti-inflammatory cytokines, neurotrophic factors, learning and memory abilities, Downregulated CysLT2R expression and ERK1/2 phosphorylation.	Zhao et al., 2020
MSCs-Exo	Rats	Stroke treatment	Neuritis remolding and Functional recovery, downregulation of connective tissue growth factor and upregulation of mir-133b after exosomes treatment	Xin et al., 2013b
MSCs-Exo	Mice/*in vitro*	Brain Hypoxic ischemia	Exosome treatment confers neuroprotection by reducing neuronal apoptosis and neuroinflammation, upregulated miR-21a-5p and its target gene was Timp3.	Xin et al., 2020
MSCs-Exo	Rats/*in vitro*	Ischemic stroke	Exosome treatment increased angiogenesis and neurogenesis in a stroke rat model	Xin et al., 2013a

### Mechanism of Exosome-Mediated Stroke Treatment

Exosomes’ brain disease–prevention characteristics are reported by a number of studies (Long et al., 2017; Zhang et al., 2017). Exosomes mainly protect ischemic brain by Li et al. (2020a) improving the microenvironment and mediating immune response, (McCann and Lawrence, 2020) inhibiting brain cell apoptosis and activating biogenesis (Cunningham et al., 2020), inducing vascular remolding and regeneration, and (Dabrowska et al., 2019) alleviating inflammation.

**FIGURE 2:**
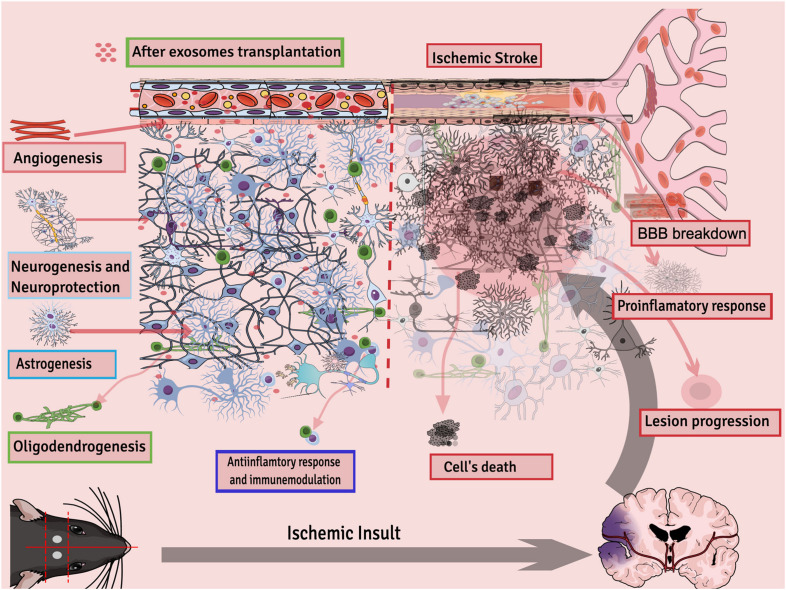
Different processes regulated by exosomes after ischemic stroke.

Exosome therapy decreases inflammation in the mouse brain and reduces infract volume and edema by reducing ROS, TNF-α, and NMDAR1 expression (Kalani et al., 2016). In a glutamate-induced nerve injury model, exosomes protected brain tissues by activating the release of cytokines and growth factors mainly by activating the phosphatidylinositol 3-kinase (PI3K)/Akt pathway (Wei et al., 2016). MSCs-Exo had a neuroprotective effect on hypoxic-ischemia injury in newborn mice, and this outcome was partly because of a decrease in neuron apoptosis and neuroinflammation. Within the injured brain, the miR-21a-5p was upregulated in neurons and microglia via uptake of MSCs-Exo (Xin et al., 2020). IV injection of exosomes improved the brain microenvironment by inducing vascular remodeling and promoting regeneration of damaged neurons in a stroke rat model (Han et al., 2018). MSCs-Exo enhanced motor function, learning, and memory abilities of rats after 7 days of middle cerebral artery occlusion (MCAO). Furthermore, an increase in production of anti-inflammatory cytokines and growth factors and a decrease in pro-inflammatory factors was found in both the hippocampus and cortex of the ischemic region as well as in OGD-treated microglia cells. Western blotting analysis results confirmed that CysLT2R expression and ERK1/2 phosphorylation were downregulated both *in vivo* and *in vitro* (Zhao et al., 2020). White blood cells, especially polymorphonuclear neutrophils, appear to be the key leukocyte population in the mediation of the neuroinflammatory response. Exosomes derived from hMSCs were studied to confer neurological recovery after focal cerebral ischemia in rodents by depleting polymorphonuclear neutrophils, especially monocytes and lymphocytes (Wang C. et al., 2020). Exosomes from MSCs were reported to reduce neuroinflammation after cortical injury in the aged brain of monkeys. Results show that recovery in exosome-treated aged monkeys was fast and efficient compared with aged control vehicle monkeys. Moreover, exosome treatment after injury is associated with greater densities of ramified, homeostatic microglia along with reduced pro-inflammatory microglial markers (Go et al., 2020). In general, exosomes’ potential therapeutic activities for stroke can be summarized as neuroprotection; reduced inflammation; immunomodulation; stimulation of new synapse formation; and activation of neurogenesis, astrogenesis, angiogenesis, and white matter remodeling (Figure 2).

### Exosome-Mediated Stroke Treatment: Pros and Cons

Exosome pros for the treatment of stroke is studied enormously (as discussed). An ongoing clinical trial (NCT03384433) aims to investigate exosome safety and potential for ischemic stroke. The main aim of this trial is a safety check, i.e., to check adverse effects within 12 months of exosome therapy, and the secondary goal is to find out the efficacy of improvement in modified ranking score, i.e., measure the degree of disability in stroke patients after 12 months. Results of the study are yet to be announced. The pitfalls of exosome applications are that it can induce neurodegenerative disorder, autoimmunity, viral infections, or the spread of cancer, as they can transmit in between cells and deliver their contents. Cancer cell–derived exosomes were immune-suppressive and had low expression levels of tumor suppressor miRNA-15a, oncogenic protein, and cytokines, and it increased cancer progression (Roccaro et al., 2013; Wen et al., 2016). Besides this, the contents and function of exosomes may depend on donor cells or on metabolic activities of receipt cells, which makes the process of exosome therapy problematic (Wiklander et al., 2015). Exosome proapoptotic and pathological communications were studied in ischemic heart injury in obesity/diabetes mellitus. Diabetic serum exosome injection to the non-diabetic heart caused poorer cardiac function recovery, larger infract size, and increased apoptosis of cardiomyocytes. It was confirmed through various analysis that miRNA-130b-3p was responsible for the cardiotoxicity, and its direct downstream targets were AMPKα1/α2, Birc6, and Ucp3 (Gan et al., 2020). Microglia-derived exosomes were found to mediate neuroinflammation (Kumar et al., 2017) and Alzheimer’s disease propagation (Saeedi et al., 2019). Moreover, preclinical data show that optimum dose is an important indicator for neurological outcomes after stroke (Moniche et al., 2017); hence, exosome time and dose-dependent studies are of utmost importance for better and safe clinical outcomes. Exosomes’ high dose was found to be detrimental for neurons although low doses show neuroprotective effects (Venugopal et al., 2017). Another study demonstrates that a low dose of exosomes was productive in ischemic conditions although, during administration of high doses, exosomes were mostly detected in the lungs or liver (Bian et al., 2014; Moon et al., 2019; Williams et al., 2020). A dose-dependent study of exosomes for stroke therapy reveals that low doses (50–100 μg) had comparatively better outcomes. At least 50 μg exosomes were necessary for subcortical stroke recovery in rats and cell proliferation in OGD conditions although *in vivo* analysis of low dosage groups showed better functional recovery compared with high dose–treated animals (Otero-Ortega et al., 2020). An exosome high dose can be detrimental and can produce negative effects; therefore, optimum effective dosage studies are mandatory.

Furthermore, it is believed that exosomes function because of miRNAs although it should be kept in mind that some miRNAs are involved in cancer pathogenesis (Van Roosbroeck and Calin, 2017). In addition, off-target effects, short half-life, tracking procedures, and clinical-level large scale production are some basic limitations of exosomes to be addressed. Subsequently, scientists are engineering native exosomes to overcome the natural limitations that we discuss in another section.

## Exosomes’ Natural Brain Targeting and Homing Abilities

Recently, various invasive and newly developed non-invasive methodologies based on overcoming the impeding action of the impermeable BBB and targeting the required disease sites of the brain have been explored. Researchers have put all their efforts toward finding therapeutic agents that can effectively target the brain. Traditional therapeutic agents do not effectively penetrate to the brain because they have to pass through four main barriers, including the BBB, blood brain tumor barrier, blood-cerebrospinal fluid barrier, and efflux protein (Khan et al., 2018). Exosomes have recently been investigated in many studies as a suitable alternative for the shortcomings of traditional agents due to their biological compatibility and particularly small size (Arrighetti et al., 2019) for brain targeting (Lapchak et al., 2018; Qu et al., 2018). Experimental studies provide new insight that exosomes and their cargo play important roles in nerve regeneration, synaptic function, plasticity, immune response, and exosome-mediated intercellular communication, contributing to brain reconstruction (Qing et al., 2018; Liu et al., 2019; Branscome et al., 2020). Exosomes’ BBB crossing ability opens new getaways to the CNS, targeting the treatment of various neurodegenerative disorders, such as stroke, Alzheimer’s disease, tumors, and autoimmune diseases (Abbott et al., 2006; Andreone et al., 2015). Luciferase-carrying exosomes can cross the BMEC monolayer under stroke mimicking inflamed condition, while not under the healthy normal conditions. Furthermore, the results show that exosomes were internalized by BMECs via endocytosis (Chen et al., 2016; Salunkhe et al., 2020). This study reveals the capability of exosomes in brain targeting by exploring their endocytic uptake via their interaction with BBB cells. Blood exosomes’ brain targeting ability was investigated *in vivo* in nude mice by injecting DiD-labeled blood exosomes through IV administration. By taking near-infrared fluorescence images at different times, it was found that exosomes were accumulated specifically in the brain between 1 and 10 h after injection, whereas the fluorescence intensity was at its peak about 4–8 h after administration of exosomes (Qu et al., 2018). MSCs-Exo were labeled with gold nanoparticles as a labeling agent to check exosome migration and brain-homing abilities. Neuroimaging results show that exosomes exactly targeted and accumulated in pathologically relevant murine model brain regions up to 96 h while in healthy controls up to 24 h (Perets et al., 2019). Exosomes can be used to deliver dopamine or drugs to the brain, and brain distribution can be increased (Yang et al., 2015; Qu et al., 2018). Exosomes are specifically internalized by microglia cells through the macro-pinocytosis pathway in a mix brain cells culture (Fitzner et al., 2011). Hence, exosomes have natural brain-homing abilities, making them suitable for brain disorders.

## Bioengineering of Exosomes for Brain Targeting

**FIGURE 3:**
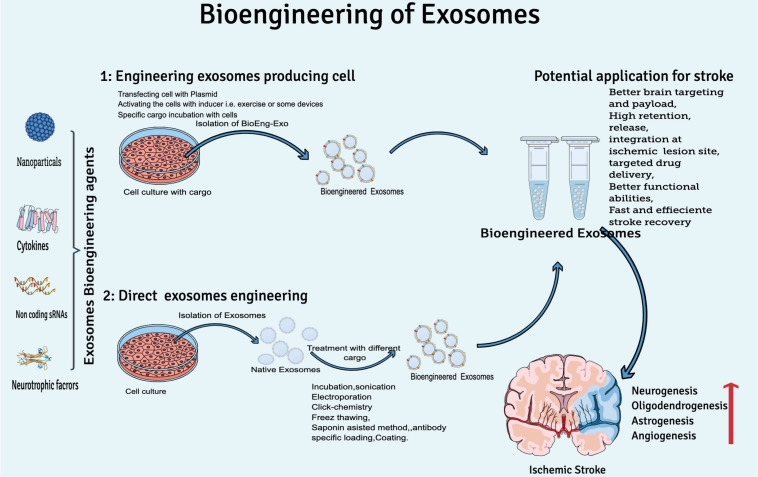
Overview of exosome bioengineering for better ischemic stroke therapy. Two basic approaches used for exosomes engineering are (1) engineering exosome-producing cells (transfecting, activating, or incubation of cargo with cells) and (2) direct exosome engineering (by loading or decorating specific cargo to exosomes after isolation).

Considering the natural therapeutic abilities of exosomes for clinical translation yet the rapid clearance of exosomes from the human brain, researchers are focusing on bioengineering of exosomes to increase concentration on target sites, circulation time, and half-life in the body. At least two conditions should be met while engineering exosomes for brain targeting: (I) BioEng-Exo must not interact with the physiological barriers, which could limit their diffusion except the plasma membrane of the target cell, and (II) exosomes should be engineered to preferably target cell types relevant to disease pathogenesis, e.g., rabies virus glycoprotein (RVG) is used to engineer exosomes specifically for CNS disease. RVG is a 29-amino-acid peptide that specifically binds to neural cells’ nicotinic acetylcholine receptor (Ferrantelli et al., 2020). T7 peptides, that target the cellular transferrin receptor in the brain has been successfully used for targeting glioblastoma in the brain (Kim G. et al., 2020). In general, exosome engineering can be done by two basic methodologies: by either engineering exosome-producing cells or direct exosome modification (Figure 3).

During bioengineering of exosome-producing cells, mainly two approaches are practiced by scientists. Either the exosome-producing cell is incubated with cargo (a specific drug or other desired solution) or by genetic transfection of the parent cell (manipulation of the cell by plasmid-containing miRNA, siRNA, or pDNA) (Tanziela et al., 2020). Production of BioEng-Exo in this way is challenging because it is very time-consuming, and sometimes the required physical and chemical conditions are not favorable for viable cells. That is why scientists mostly prefer post exosome modification techniques.

Direct exosome bioengineering is done after isolating and cleaning exosomes from a cell’s ordinary liquid, such as culture medium, serum, and other cell debris. Postproduction exosome bioengineering is a feasible method because exosomes are non-living structure, so it is easy to apply the condition of choice, and high yield can be loaded onto exosomes compared with the cell-based modification approach. Direct exosome engineering can be done by incubation, sonication, electroporation, antibody-specific loading, the freeze-and-thaw method, and the saponin assist method (Tanziela et al., 2020). Through these modification techniques, researchers either modify the surface or contents of exosomes.

### Exosome Surface Modification

Despite recent progress unlocking exosomes’ secrets, advancements in surface modification techniques are mandatory for future clinical translation. Through surface manipulation strategies, exosome circulation, targeting, and stay time can be increased, and it can be done by both active (sonication, electroporation, etc.) or passive (incubation, diffusion etc.) techniques.

Scientists have coated exosomes with polyethylene glycol, which is a hydrophilic polymer, and increased the circulation time of nanoparticles (Suk et al., 2016). The PEGylation technique increases the circulation time of exosomes and reduces non-specific interaction with cells while improving interaction with EGFR-expressing cells. The PEGylation approach was employed by incubating the polyethylene glycol with exosomes 1:1 for 2 h at 40°C to enhance Neuro2A cell–derived exosome specificity and circulation time (Kooijmans et al., 2016). Exosomes targeted specifically toward the brain were achieved by engineering the exosome-producing cells to express an exosomal protein, lysosome-associated membrane protein 2 (Lamp2b), attached to the neuron-specific RVG peptide (Alvarez-Erviti et al., 2011). The glycosylation motif was introduced at various positions of the Lamp2b protein, which prevented the exosome-targeting peptide from degradation as well as stabilized targeted delivery of therapeutic exosomes (Hung and Leonard, 2015). Special membrane-penetrable agents can be stacked to exosomes by incubating the small compound with exosomes under specific conditions (25–37°C). Exosomes were decorated with a brain-targeting peptide (low-density lipoprotein) by the incubation approach for directing the exosome payload toward the glioma site in the brain. The peptide contained an ApoA-1 mimic sequence that enabled its linkage to exosomes by simple incubation (Ye et al., 2018). Click chemistry, originally introduced in 1999, is a reliable, simple, fast, and highly efficient technique for bioconjugation of small and macro molecules to the exosome surface (Hein et al., 2008; Smyth et al., 2014). The targeting ability of exosomes can be improved by surface functionalization. The peptide c(RGDyK), having high affinity to integrin αVβ3 in reactive cerebral vascular endothelial cells, after ischemia especially, was conjugated to the exosome surface through click chemistry reactions. Tail-vain injection of cRGD-Exo specifically targeted the lesion region of a mouse model of cerebral ischemia as well as entered the neuron, microglia, and astrocytes (Tian et al., 2018). Another group conjugated a neuropilin-1–targeting peptide to exosomes through click chemistry for glioma therapy (Jia et al., 2018). To cross the BBB, a gold nanoparticle surface was modified with brain-targeting exosomes. The unique brain-targeting ability of exosomes enabled the gold nanoparticles to cross the BBB while their binding to brain cells was examined under laminar flow conditions (Perets et al., 2019). GFP-CD63–labeled exosomes from the primary neuron are taken up by neurons preferentially, whereas those from a neuroblastoma cell line bind equally to astrocytes (Chivet et al., 2014). All the abovementioned studies confer exosomes with great potential to be polished and developed as effective, safe, and precise agents for CNS neurodegenerative disorders.

### Exosome Cargo Modification

Desired cargo can be loaded onto exosomes by pre- or post-isolation modification methods. The most simple and straightforward technique for exosome cargo loading is incubation by which the desired cargo is incubated with exosome-producing cells or with exosomes. Exosomes are promising candidates for targeted delivery of various material to specific cells because of the lipid bilayer membrane decorated with multiple ligands and receptors that can interact with target molecules (Tang et al., 2020). Exosomes were loaded with catalase or quercetin by the incubation technique for neuroprotection *in vivo* and *in vitro* (Haney et al., 2015; Qi et al., 2020). Proteins were loaded onto exosomes by an optically reversible protein–protein interaction method, which significantly increased loaded protein levels in recipient cells (Yim et al., 2016). Another group manipulated exosome cargo by loading them with macromolecules, such as proteins and ribonucleoprotein, for cellular delivery (Zhang X. et al., 2020). MiR-210 loaded exosomes were produced by the incubation technique for targeted delivery of miR-210 to the ischemic stroke lesion (Zhang H. et al., 2019). Exosomes were produced through genetic modification for cerebral protection against deep hypothermic circulatory arrest. MSC culture was transfected with pre-miR-214 containing lentivirus vectors, which significantly increased the miR-214 expression in the extracted exosomes (Shi et al., 2020). Exosomes loaded with curcumin strongly suppressed the pro-inflammatory cytokines and cellular apoptosis in the stroke lesion area and activated microglia cells (Tian et al., 2018). Moreover, physical treatment techniques, such as sonication, electroporation, extrusion, surfactant treatment, and dialysis, are employed for loading of exosomes with specific cargo for brain targeting (Alvarez-Erviti et al., 2011; Haney et al., 2015; Fu et al., 2020).

Besides the stated exosome bioengineering methods and examples for the brain, some of the latest research on exosome engineering techniques for brain targeting (specifically for ischemic stroke therapy, Table 2) are discussed in the next sections, in which exosomes have been bioengineered by special agents for ischemic stroke therapy (Figure 3).

**TABLE 2:** BioEng-Exo for stroke.

Name	Aim	Engineering agent	Method of engineering	Result	References
Golden Exosomes	Migration and homing abilities of exosomes in the brain	Gold nanoparticle	Incubation of GNP with exosomes and collect golden exosomes by ultracentrifugation	Tracked the exosomes’ brain-homing abilities in different brain disease models	Perets et al., 2019
Magnetic Nanovesicle	Checking IONP-NVs ability for targeting ischemic lesion	IONP	Treat MSCs with IONPs and isolate NVs through ultrasonication and centrifugation	Enhanced angiogenesis, growth factors, antiapoptotic and anti-inflammatory and ischemic recovery effects	Kim H.Y. et al., 2020
cEPC-Exosomes	Explore the effect of exercise on cEPC-EXs for stroke	Exercise	Moderate treadmill exercise for 4 weeks before MCAO	Protective effect on the brain against MCAO by increasing EPC-EXs-miR-126	Wang J. et al., 2020
Exo-pGel	Establish implantation strategy of exosomes for injured neurons	Hydrogel	By injecting the exosomes to prepared pGEL and incubating them overnight	Prolonged retention of exosomes for nerve tissue recovery	Li et al., 2020b
IFN-γ-hNSC-Exo	To check IFN-γ-hNSC-Exo vs. hNSC-Exo potential for stroke	Interferon gamma cytokine	By treating exosomes producing cells with IFN-γ	Increase cell proliferation and survival *in vitro* as well speed up stroke recovery efficiently	Zhang G. et al., 2020
RVG-Exosomes	Delivery of siRNA to brain.	RVG peptide and siRNA	Expressed Exo-membrane protein Lamp2b attached to RVG on exosomes producing cells and loaded exosomes with siRNA by electroporation	Specifically delivered GAPDH siRNA to neurons, microglia, and oligodendrocytes and knocked down BACE1 gene	Alvarez-Erviti et al., 2011
MSCs-secretome	Find out IL-1α-primed MSC-derived secretome effect on stroke	Interleukin-1α	MSC culture was treated with IL-1α then incubated together for 24 h, and secretome was collected by filtration.	Improved nest building and neurological score and reduced 30% stroke lesion volume	Cunningham et al., 2020
RVG-circSCMH1-EVs	Explore potential of circRNAs using EVs as targeted delivery system	RVG peptide and Circular RNA SCMH1	Transfection of HEK293T cells with plasmids encoding the GNSTM-RVG-Lamp2b-HA and the circSCMH1 plasmids followed by EVs purification	Improved functional and behavioral recovery post stroke both in mice and monkeys, enhance neural plasticity by binding to MeCP2 gene	Yang L. et al., 2020
RGD-Exosomes	Safe and efficient delivery of miR-210	c(RGDyK) peptide and miR-210	Incubation of RGD-Exo with miR-210 for 1 h at 37°C	Enhance expressions of integrin β3, vascular endothelial growth factor (VEGF) and CD34 and mouse stroke survival rate	Zhang H. et al., 2019
RVG-Exosomes	Delivery of miR-124 to infract size	RVG-Peptide and miR-124	Electroporation	Promote neurogenesis and functional recovery	Yang J. et al., 2017
NGF@ExoRVG	Delivery of nerve growth factor to ischemic region	Neurotrophic factor (NGF)	Transfection of cells cultured with pcDNA3.1(-)-RVG-Lamp2b and pCI-neo-NGF plasmids for exosomes production	NGF delivery to ischemic region that reduced inflammation and promoted cell survival.	Yang J. et al., 2020

### Nanoparticle

Advanced studies have demonstrated that exosomes Possess equivalent therapeutic potential of derived stem cells for ischemic stroke treatment (Zhang Z.G. et al., 2019). However, the most serious drawback of using exosomes is the poor targeting of the ischemic lesion in the brain. Magnetic nanovesicle (MNVs) derived from iron oxide nanoparticle-harbored MSCs increased the targeting of the ischemic region in the brain with the help of an external magnetic field by magnetic navigation. Magnetic navigation increased the exosomes’ ability to target the ischemic lesion by 5.1 times. Moreover, the MNVs considerably decreased the infract volume and improved motor function as well as promoted an anti-inflammatory response, angiogenesis, and anti-apoptosis in the ischemic brain lesion (Kim H.Y. et al., 2020). The exosomes are able to cross the BBB, but their migration and brain-homing abilities were yet to be discovered. A research group developed a method for longitudinal and quantitative *in vivo* neuroimaging of exosomes while combining exosomes with gold nanoparticles. Exosomes were tracked in different brain conditions, such as ischemic stroke, Alzheimer’s disease, etc. The results show that exosomes accumulated only in the diseased brain and were gradually cleared from the healthy brain, and the special protein structure of exosomes was important for their precise and extended accumulation in the brain (Perets et al., 2019). Glucose-coated gold nanoparticles were used in a mouse model of brain ischemia for non-invasive neuroimaging and tracking of exosomes, which helped to find the optimal administration route and size parameter (Betzer et al., 2017). Treatment and diagnosis for many neurological diseases are hindered by the inability of theranostic agents to cross the BBB (Moura et al., 2019). Following the concept of the nature biotechnology group, who developed exosomes as gene therapy vehicles for specific brain targeting (Alvarez-Erviti et al., 2011), exosome-coated gold nanoparticles were shown to induce targeted delivery to brain cells by increasing the permeability of nanoparticle to cross the BBB (Khongkow et al., 2019).

### Cytokines

Cytokines are soluble glycoproteins that play an important role in the pathophysiology of stroke. The loss in between pro-inflammatory and anti-inflammatory cytokines occurs after stroke and affects infarct size and functional outcome (Doll et al., 2104; Klimiec-Moskal et al., 2020). A study compared the role of exosomes derived from interferon gamma (IFN-γ)-stimulating stem cells and control exosomes for treating ischemic stroke and found that IFN-γ preconditioning did not affect the secretion, but significantly altered the functional abilities of exosomes. Moreover, IFN-γ exosomes increased cell proliferation and cell survival as well as decreased cell apoptosis *in vitro* while exerting therapeutic effects *in vivo* in an ischemic rat model (Zhang G. et al., 2020). IFN-γ-exosomes have been used for treating neurodegenerative disorders, i.e., multiple sclerosis. IFN-γ-stimulated exosomes reduced demyelination and decreased neuroinflammation in a mouse model (Riazifar et al., 2019). The effect of tumor necrosis factor alpha and interleukin-1β cytokines was evaluated on the release and molecular composition of astrocyte-derived exosomes, and results confirm that TNFα- and IL-1β-treated astrocyte-derived exosomes were rich in miR-125a-5p and miR-16-5p that target proteins involved in neurotrophin signaling. In addition, they observed that cytokine-treated exosomes decrease neuronal NTKR3 and Bcl2 expression (Chaudhuri et al., 2018). Inflammatory cytokine IL-1β that regulates the brain’s injury inflammatory response was injected into brain; a striatal injection of IL-1β promoted an influx of Ly6b+ leukocytes to the lesion site as well as increasing circulating exosome levels in the plasma of mice compared with controls. IL-1β also induced the release of astrocyte-derived exosomes that rapidly crossed the BBB (Dickens et al., 2017). LPS/IFNγ-treated microglia-derived exosomes reduced brain tumor and promoted brain homeostasis recovery (Grimaldi et al., 2019). IL-4-polarized BV2-exosomes promoted angiogenesis in an MCAO model of ischemic stroke (Tian et al., 2019). IL-1-primed MSC-secreted conditioned medium was assessed to promote recovery after stroke. IL-1α-primed MSC-derived conditioned medium treatment led to ∼30% reduction in lesion volume and improved behavioral outcomes and neurological score in a mouse model of stroke (Cunningham et al., 2020). In another study, IL-4 and lipopolysaccharide polarized microglia BV2 cells were investigated for pro-angiogenesis effects. IL-4-polarized cells increased the tube formation of endothelial cells by secreting exosomes, and the miRNA-26a profile was higher compared with the LPS-polarized group (Tian et al., 2019). IL-6, a proinflammatory cytokine that can promote the prosurvival signaling pathway, preconditioned in neural stem cells to reduce ischemic injury in a mouse model of stroke (Sakata et al., 2012).

### Small Non-coding RNAs

The field of exosome research is still in its infancy, particularly in studies of CNS diseases. The endothelial cells of brain capillaries form extremely tight junctions creating the BBB, which restricts the entry of all small molecules that are insoluble in lipids (90–98%) to the brain (Yang J. et al., 2017; Blanchard et al., 2020; Su et al., 2020). Yang et al. used exosomes for the delivery of circular RNA to the ischemic region of stroke. They specifically targeted neuronal cells by expressing RVG peptide on exosome membranes and used this RVG-Exo as a cargo delivery system for Circ-SCMHI RNA. RVG-circSCMH1-EVs improved neuronal plasticity by binding to MeCP2 and increased its downstream gene expression (Mobp, Igfbp3, Fxyd1, and Prodh), which maintains brain function. IV injection of RVG-CircSCMH1-Exo improved motor recovery, digit movement, and functional recovery in both rodents and non-human primate models. They suggested that RVG-CircSCMH1-Exo could have wider therapeutic potential window compared with current therapies as they could be administered 24 h after stroke onset (Yang L. et al., 2020).

MiR-124 is well known for its proneuronal role in both the developing and mature brain. Exosomes loaded with miR-124 promote cortical neural progenitors to obtain neuronal identity and confer recovery after ischemia by robust cortical neurogenesis (Yang J. et al., 2017). SiRNA was delivered by harnessing exosomes as shuttle servers. IV injection of exosomes delivered siRNA to neurons, microglia, and oligodendrocytes in the mouse brain. Exosome-mediated delivery of siRNA knocked downed the BACE1 gene, and the inhibition of BACE1 significantly decreased β-amyloid levels in the brain of wild-type mice (Alvarez-Erviti et al., 2011). Consecutively, another group used exosomes as an siRNA carrier assuming it as a possible key step toward siRNA clinical application (van den Boorn et al., 2011). MiRNA-210 holds great potential to improve angiogenesis for brain tissue repair after ischemia. Upregulation of miRNA-210 improved functional recovery after stroke. MiRNA-210 was delivered to the ischemic lesion by conjugating exosomes with c(RGDyK) peptide and then loading with miRNA-210. RGD-Exo miR-210 increased the miR-210 level at the ischemic site as well as upregulated integrinβ3, vascular endothelial growth factor, and CD34, thus increasing the animal survival rate (Zhang H. et al., 2019). Exosomes were utilized for HMGB1-siRNA delivery for treatment of ischemic stroke. Results indicate that exosomes combined with HMGB1-siRNA decreased HMGB1, TNF-α, apoptosis, and infract volume, showing potential for recovery of ischemic stroke (Kim et al., 2019). All these studies indicate that exosomes loaded with small RNAs can enormously alter its abilities for ischemic stroke treatment.

### Neurotrophic Factors (NTFs)

Neurotrophic factor are studied as a neuroprotectant in neurovegetative disorders (Lindholm and Saarma, 2010; Houlton et al., 2019). They control neural stem cell differentiation, which is responsible for the recovery of neural cell function and vessel damage due to ischemic stroke (Abe, 2000). NTFs are emerging as a viable repair therapy in stroke, and they are recognized for their multifaceted neuroprotective role after ischemia (Ramos-Cejudo et al., 2015). However, NTF clinical application is yet not applicable because of the lack of an efficient systemic delivery approach to the ischemic region. Engineered exosomes were used for the delivery of nerve growth factor (NGF) to the ischemic cortex of mouse brain. HEK293 cells were incubated with NGF-Exo different quantities for 4 h, and the mRNA and NGF proteins were checked through qPCR, which showed significant increases in both mRNA and NGF protein expression levels, suggesting that engineered exosomes could successfully deliver NGF mRNA and protein to target cells *in vitro*. A photothrombotic ischemia model was used, and Dil-labeled exosomes were injected through the tail vein 24 h postischemia. Results show that a remarkable Dil-positive ischemic region was detected in both RVG and NGF-EXO compared with the control exosome group as well as the Dil signal in the ischemic region overlapped with the markers of neuron, astrocyte, and microglia, suggesting that exosomes were taken up by these cells (Yang J. et al., 2020). Brain-derived neurotrophic factor (BDNF) has been investigated enormously for conferring neuroprotection and anti-inflammatory properties. BDNF was encapsulated inside naïve exosomes and delivered to the brain against brain inflammation through IV administration to mice (Donoso-Quezada et al., 2020). MiR-206 knockdown exosomes attenuated early brain injury by upregulation of BDNF levels after exosome treatment (Zhao et al., 2019), which gives us an idea that bioengineering exosomes with BDNF could have a positive outcome for brain disorders, including stroke. However, the collaboration of both exosomes and NTFs has not been done for stroke therapy in depth; indeed, it is a worthwhile field to explore in the future.

## Conclusion and Future Prospective

Exosomes are secreted by almost all cell types and contain lipids, proteins, and nucleic acids of the origin cells. They are at the forefront of clinical success in many research areas. In the past few years, the effectiveness of exosomes has been enormously studied as being the best fit for brain targeting because of their native abilities to cross the biological barriers into the brain. However, there is evidence that exosomes get cleaned up from the brain quite quickly, and in a short time period, very low amounts of exosomes can be seen in the brain. Therefore, scientists have focused on bioengineering exosomes to increase their circulation half-life, increase their stay at the disease site, direct exosomes to target cells, and use them for targeted delivery of therapeutic molecules or for regenerative medicine. Scientists have BioEng-Exo with lots of agents (as discussed in the review) for targeted stroke therapy, but still, this field is in its infancy, and lot yet needs to be done. In preclinical studies, promising results are obtained, but the effect is still unknown in humans. Many studies are been carried out, but mostly *in vitro*, in which the culture condition may affect the exosomes’ biochemical and biophysical features; therefore, future studies are needed to better understand the physiological effects of these engineered exosomes on human health. Moreover, the bioactive molecules loaded in exosomes are extremely small and low in quantity; therefore, there is need of efficient Exo-specific recognition tools in recipient cells. Immune tolerance, potency, and toxicity of these exosomes need to be explored in *in vivo* studies. Moreover, further work is needed on stabilization, and optimization of these BioEng-Exo techniques as well as isolation, high scalable production, purity, and stability of relevant exosomes is mandatory for future potential clinical success.

## Author Contributions

HK conceived, designed, and wrote the manuscript. J-JP and YL provided critical revision and helped in the analysis of manuscript. G-YY and ZZ contributed to the discussion of ideas, helped in the correction, and proofread the manuscript. All the authors contributed to the article and approved the submitted version.

## Conflict of Interest

The authors declare that the research was conducted in the absence of any commercial or financial relationships that could be construed as a potential conflict of interest.

## Abbreviations

BioEng-Exo: bioengineered exosomes
BBB: blood–brain barrier
BDNF: brain derived neurotrophic factor
BMECs: brain microvascular endothelial cells
CNS: central nervous system
EEs: early endosomes
EVs: extracellular vesicles
ESCRT: endosomal-sorting complex essential for transport
HIV: human immunodeficiency virus
ILVs: intraluminal vesicles
IFN- γ: interferon gamma
IL: interleukin
IONP: iron oxide nanoparticle
Lamp2b: lysosome-associated membrane protein 2
MSCs: mesenchymal stem cells
MVs: microvesicles
MVBs: multivesicular bodies
MCAO: middle cerebral artery occlusion
MSCs-Exo: MSCs exosomes
MNVs: magnetic nanovesicle
NTFs: neurotrophic factors
NGF: nerve growth factor
RVG: rabies virus glycoprotein.
